# Effects of small-sided games and dribbling circuit training on physical and technical skills in youth soccer players

**DOI:** 10.1038/s41598-025-31382-7

**Published:** 2025-12-11

**Authors:** Mohamed Amine Rahmoune, Okba Selmi, Souheir Bouali, Anissa Bouassida, Dan Iulian Alexe, Monira I. Aldhahi, Adin Marian Cojocaru, Mirela Ştef, Cristina Ioana Alexe

**Affiliations:** 1 High Institute of Sports and Physical Education of Sfax, Sfax, Tunisia; 2 High Institute of Sports and Physical Education of Kef, El Kef, Tunisia; 3 Research Unit: Sportive Sciences, Health and Movement, El Kef, Tunisia; 4 Laboratory of Analysis and Expertise of Sports Performance, Institute of Science and Technology of Physical and Sports Activities, AbdelhamidMehri, University Constantine, Constantine, Algeria; 5 Department of Physical and Occupational Therapy, “Vasile Alecsandri” University of Bacau, 600115 Bacău, Romania; 6 Department of Rehabilitation Sciences, College of Health and Rehabilitation Sciences, Princess Nourah bint Abdulrahman University, P.O. Box 84428, 11671 Riyadh, Saudi Arabia; 7 Faculty of Physical Education and Sport, Spiru Haret University, 041905 Bucharest, Romania; 8 Departament of Physical Education, Sport and Physical Therapy, University of Oradea, 410087 Oradea, Romania; 9 Department of Physical Education and Sports Performance, “Vasile Alecsandri” University of Bacău, 600115 Bacău, Romania

**Keywords:** Small-sided games, Hoff circuit, Physical performance, Technical skills, Soccer players, Health care, Physiology, Psychology, Psychology

## Abstract

This study investigated the comparative effects of small-sided games (SSG) and Hoff circuit (HC) training on physical, technical, and psychophysiological parameters in highly trained youth soccer players. Twenty-six youth players from a professional team were randomly allocated to either an SSG group (n = 13, age = 17.53 ± 0.49 years) or an HC group (n = 13, age = 17.54 ± 0.5 years). The six-week intervention comprised two additional training sessions per week alongside regular team training. Pre- and post-program assessments evaluated physical performance (Vameval test, 5-Jump Test, 10 m and 30 m sprints, and Illinois agility test), technical skills (passing accuracy, possession, interceptions, tackling, and heading). Perceptual responses (RPE and PACES) were evaluated after each session. The HC protocol involved a 290 m dribbling circuit performed at 90–95% HRmax, whereas the SSG emphasized ball possession and tactical gameplay under game-like conditions. The results showed that both training modalities significantly improved maximum aerobic speed (MAS) (*p* < 0.05). However, the SSG group demonstrated superior improvements in sprint performance (10 m and 30 m; *p* < 0.01) and agility (Illinois test; *p* < 0.01) compared to the HC group. SSG training was also more effective in enhancing technical skills, particularly passing accuracy (*p* < 0.05) and interceptions (*p* < 0.05). No significant between-group differences were observed in heart rate responses (*p* > 0.05) or RPE (*p* > 0.05). Notably, SSG participants reported significantly higher enjoyment levels (*p* < 0.05), suggesting enhanced motivation and training adherence. SSG training offers superior benefits for sprint performance, agility, technical skill development, and player enjoyment compared to circuit-based training in youth soccer players. These findings support the integration of SSG as a primary training methodology for coaches seeking to optimize athletic performance, technical proficiency, and player engagement in youth soccer development programs.

## Introduction

Soccer is a high-intensity sport that requires players to perform a complex combination of sprints, rapid directional changes, and jumps, imposing significant physiological, technical, and cognitive demands on athletes^1^_._ Given the intermittent nature of soccer, players must develop a well-rounded athletic profile that encompasses endurance, agility, speed, strength, and technical mastery. The ability to sustain high-intensity efforts while maintaining technical precision and tactical awareness is crucial for optimizing the performance. Consequently, structured training programs that replicate the physical and cognitive challenges of competitive matches are essential for enhancing players’ capabilities^2^.

Recognizing this necessity, coaches and sports scientists have increasingly focused on developing training methodologies that closely simulate real game conditions^3,4^. Traditionally, conditioning programs in soccer have emphasized on broad physical fitness, such as strength, sprint capacity, and aerobic endurance, frequently through interval work, resistance training, or running drills, with little focus on technical or tactical aspects^2^. However, this physically oriented approach often fails to reflect the complex and multifaceted demands of real match play. Indeed, match performance requires continuous interaction between physical execution, tactical decision-making, and technical precision, which traditional methods do not fully reproduce. In response to these limitations, Modern training approaches aim to integrate technical, tactical, and physiological components to maximize match-specific performance^5^. Among these approaches, Small-sided games (SSGs) and Hoff circuit (HC) training have gained recognition for their ability to develop soccer-specific skills through match-like, high-intensity activities^5,6^. HC training uses systematic circuit-based exercises that combine aerobic and anaerobic efforts with ball-handling drills, simultaneously improving cardiovascular fitness, muscular endurance, and technical abilities^6–8^. Through the integration of sport-specific exercises into an endurance-based program, HC training enhances muscular endurance and cardiovascular fitness, potentially improving performance during games^6^. Additionally, its intensity-driven structure produces heart rate responses that are similar to those of competition, indicating that it can successfully mimic some of the physiological demands of competitive settings^8^. Research on young players (13–16 years old) shows that HC interventions, such as a 6-week program, elicit heart rates (HR) comparable to competitive matches while enhancing repeated sprint ability, technical execution, and VO₂max^7,8^. Coaches can adjust technical complexity, volume, and intensity within this structured framework, allowing quantifiable monitoring of player progress.

Similarly, SSGs create game-like scenarios that integrate tactical, technical, and physical development^9,11^. By modifying pitch size, player numbers, or rules, SSGs generate repeated high-intensity efforts and frequent technical actions, closely mirroring match demands^10,11^. Studies indicate that SSGs significantly improve agility, horizontal jump, aerobic and anaerobic capacity, as well as engagement and motivation, promoting adherence and long-term skill acquisition^12^. For instance, 4v4 SSGs have been shown to markedly increase high-intensity running and technical involvement in U15 players^10^. Moreover, SSGs are associated with increased player enjoyment and intrinsic motivation owing to their engaging and competitive format, which can promote long-term adherence and skill development^9^. The psychological benefits of SSGs, particularly in terms of motivation and player engagement, make them an attractive option for youth training programs^2^. However, the effectiveness of small-sided games can be influenced by inappropriate task design, inadequate coach supervision, or differences in player fitness^13^. Excessive use may also limit exposure to full-pitch tactical situations^14^. Therefore, careful and balanced integration within the training program is essential to maximize benefits and minimize potential drawbacks^13^.

Although both training methods are widely used in soccer preparation, limited research has directly compared their effects on young players’ physical performance, physiological responses, and psychological engagement. Understanding how these training modalities influence fitness, motivation, and enjoyment is important for optimizing training strategies. As enjoyment is a key factor in long-term athletic development, assessing the psychological impact of these training methods is essential for designing effective youth training programs. This study aimed to evaluate and compare the effects of SSG and HC training on the physical capacities, technical performance, and psychophysiological responses of highly trained youth soccer players, providing valuable insights for coaches seeking to implement the most effective training programs. By analyzing their respective contributions to physical conditioning, skill development, and psychological engagement, this study offers a comprehensive perspective on best practices for fostering young soccer talent.

## Materials and methods

### Participants

Twenty-six youth players from a professional soccer club competing in national youth leagues in Algeria participated in the study. All participants had over 6 years of competitive soccer experience and typically trained 6 to 7 times per week, with an average training duration of 9.5 ± 1.54 h per week. The players were divided into two groups: the SSG group (SSG-G), which engaged in SSG training (n = 13, age: 17.53 ± 0.49 years, height: 177.5 ± 3.27 cm, weight: 72.76 ± 3.27 kg, body fat %11 ± 1.03), and the HC training group (HC-G), which followed an HC training program (n = 13, age: 17.54 ± 0.5 years, height: 176.84 ± 3.43 cm, weight: 71.92 ± 3.97 kg, body fat %11.23 ± 1.12). Goalkeepers were not included in the study because of their different physical training regimens. Prior to initiating the recruitment process, a sample size calculation was performed using G*Power software (version 3.1.9.4; University of Kiel, Kiel, Germany). The calculation showed that 26 players were necessary to achieve an 81.5% likelihood of correctly rejecting the null hypothesis and identifying significant differences. The players were familiar with the testing protocols, which were part of their performance assessments, and were advised to maintain consistent dietary and hydration practices throughout the testing period. Written informed consent was obtained from all players. The study was approved by the Research Ethics Committee of the High Institute of Sports and Physical Education of Kef (approval number: 013/2023; approval date: April 18, 2023) and was conducted in compliance with the ethical standards for human research as outlined in the Declaration of Helsinki.

### Study design

The study was conducted during the 2023–2024 soccer season lasting six weeks. It followed a controlled experimental design in which participants were assigned to two training interventions: Hoff Circuit (HC) and small-sided games (SSG). The study took place on the same synthetic grass training field and at the same time each day to minimize circadian effects on physiological variables. Before the start of the six-week training program, participants underwent anthropometric measurements (weight and height) and completed a progressive maximal test (Vameval) to estimate their maximal aerobic speed (MAS) and determine their maximum heart rate (HRmax), which was subsequently used to individualize training intensities. The intervention consisted of two training sessions per week, implemented alongside regular team training. Physical performance (Vameval test, 5-jump test, 10 m and 30 m sprints, Illinois agility test), technical performance (5 vs. 5 SSG metrics including passing, possession, interception, tackling, and heading), and psychological responses (rating of perceived exertion [RPE] and physical enjoyment [PACES]) were assessed before and after the program. Participants were instructed to avoid intense activity within 48 h prior to assessments, refrain from eating within 3 h of testing, and abstain from caffeine for 8 h before evaluations. To minimize potential learning effects, all participants were familiarized with the RPE, PACES, and POMS questionnaires prior to the study, and all test sessions were conducted by the same coaching staff (two physical coaches and one assistant coach) to ensure consistency.

### Training intervention descriptions

Both training protocols began with a standardized 15-min warm-up designed to prepare the participants for the upcoming physical demands. The warm-up included light jogging to gradually elevate heart rate, coordination drills to enhance motor skills, and dynamic stretching to increase flexibility and range of motion. The session concluded with four 10-m sprints to stimulate anaerobic performance. Static stretching was intentionally excluded from the warm-up to maintain muscle elasticity and readiness for high intensity activity. After the warm-up, a 3-min recovery period allowed the participants to hydrate and prepare mentally for the training. Each training method lasted 28 min, consisting of four intervals of 4 min each, followed by 3 min of passive recovery between intervals. This structure ensured that the participants could maintain high intensity throughout the training while providing adequate recovery to optimize performance gains.

**Fig. 1:**
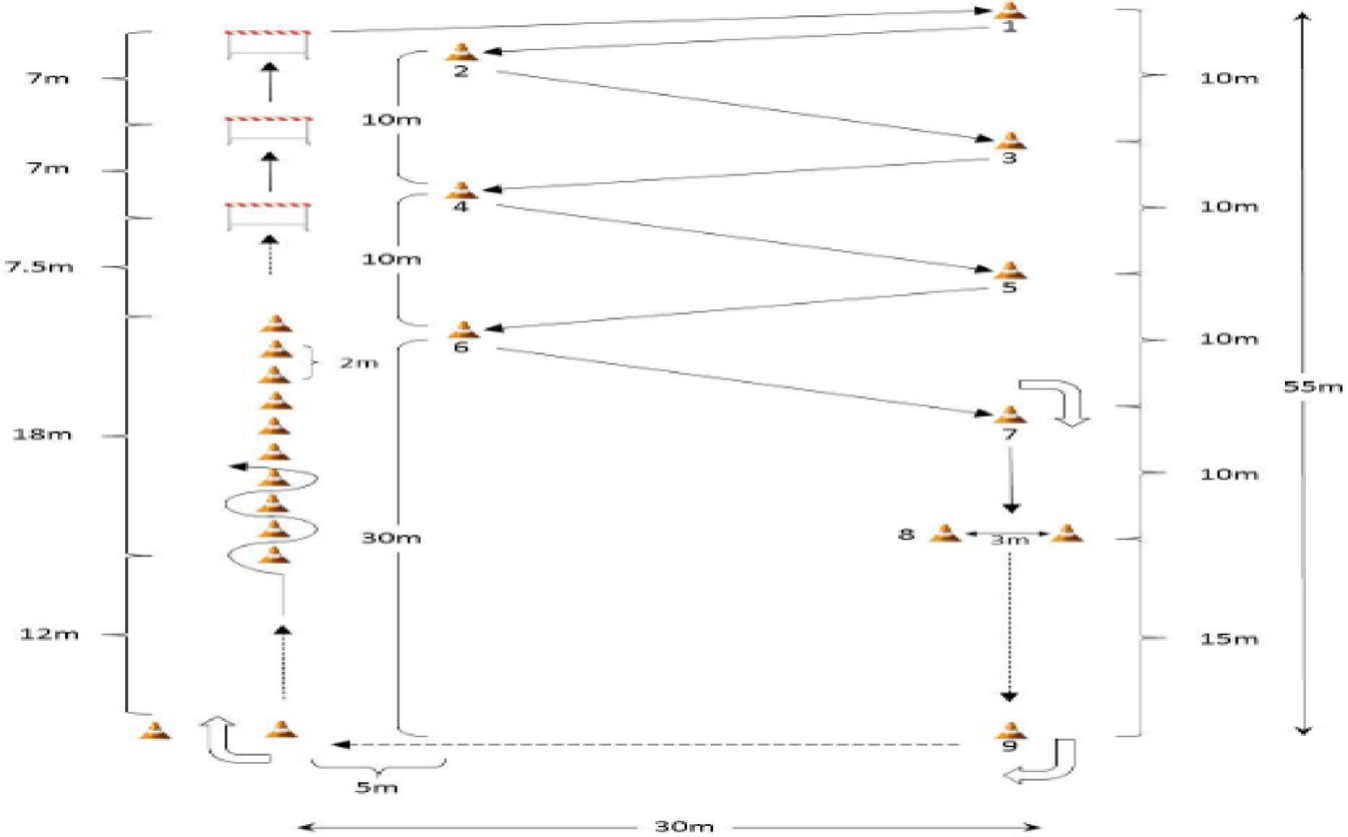
Diagram of Hoff circuit exercise.

For the Hoff circuit (HC) exercise, all players performed the protocol simultaneously, although they did not start from the same position; each player began at a different point on the circuit because the circuit was too long. Players navigated a dribbling circuit while the Polar Team Sport System ensured their HR stayed between 80 and 90% of HRmax in real time. The circuit has a total length of 51.5 m on the left side and 55 m on the right, with a width of 35 m. Players perform backward dribbling between cone 7 and the goal at Gate 8. The circuit was equipped with three hurdles (30–35 cm in height) and 22 cones, two of which marked the starting line and two served as gates for the backward running. Each lap covered a distance of 290 m. The distance from hurdle 3 to cone 1 was set at 30.5 m, with the spacing between cones (1–2, 2–3, 3–4, 4–5, 5–6, and 6–7) consistently set at 25.5 m (Fig. 1). If a player did not reach the target intensity, the evaluator verbally instructed them to increase their speed. The circuit’s length prevented any collisions or interferences between players, as faster players could overtake slower ones and continue their effort uninterrupted. The 5 vs. 5 SSG exercises were held outdoors on a field measuring 36 by 24 m, strictly following the dimensions and time constraints established in prior studies^12,15^. Coaches played an active role in motivating players to sustain a high level of intensity throughout the sessions, providing additional balls as necessary to ensure an uninterrupted play. The primary objective of the SSG was to maximize ball possession, encouraging players to develop tactical awareness and decision-making skills while maintaining control of the game. The coaches actively moved around the training area, offering support and motivation to the participants throughout the exercise.

#### Anthropometric measurements

Height and body mass were measured following standardized protocols, with measurement errors of ≤ 0.2 kg and 5 mm, respectively. A digital scale (OHAUS, Florham Park, NJ, USA) was used to record height and body mass to the nearest 0.1 cm and 0.1 kg, respectively. Body fat percentage was estimated using the four-site skinfold thickness (biceps, triceps, subscapular, and supraspinal), measured with a calibrated Harpenden caliper (Holtain Instruments, UK).

#### Physical performance assessments

Physical performance evaluations included the 5-Jump Test (5JT), Vameval test, 10 m and 30 m sprint tests, and Illinois agility test. These assessments were conducted prior to the start of the study and again after a 6-week training period. Testing was organized over two consecutive days: the first day included the sprint tests, the Illinois agility test, and the 5JT, while the second day focused on the Vameval test. The 5JT was conducted outdoors on synthetic grass in line with the methodology established by Chamari et al.^8^. The participants performed five consecutive jumps, and the total distance covered was measured in meters using a measuring tape. Two maximal 30-m sprints were performed on a synthetic turf surface, with a split time taken at the 10-m mark. Each sprint was separated by a 3-min recovery period^16^. The starting position was standardized to a stationary split stance, with the toe of the dominant foot positioned 0.5 m behind the start line. Times were measured using photoelectric cells (Brower Timing System; Salt Lake City, UT, USA). to ensure precise timing. The fastest times at both the 10-m and 30-m marks were used for analysis. The Illinois agility test was used to assess the participants’ agility^17^. The course, marked by two lines 10 m apart, had participants start lying flat with their hands by their shoulders. After a verbal cue, they sprinted to the touch line (point A), returned to the starting line (point B), and navigated the cones. They sprinted to the touch line (point C) before completing the task. Participants aimed to complete the course quickly, with errors such as knocking cones or deviating from the path, resulting in trial cancellation. Time was recorded using a photoelectric timing system (Brower Timing System; Salt Lake City, UT, USA). To assess individual maximal aerobic speed (MAS) and maximum heart rate (HRmax), the participants performed the Vameval test on a 200-m outdoor track^15^. The players ran between ten cones positioned 20 m apart, following an auditory cue. The test began at a speed of 8 km h^-1^ and increased by 0.5 km h^-1^ every minute until the players reached exhaustion. The speed achieved during the final one-minute stage was recorded as the player’s MAS. Throughout the test, HR was monitored using a Polar Team Sport System, and the highest average heart rate over a 5-s interval was noted as HRmax.

#### Technical performance

The technical performance metrics analyzed during 5 vs. 5 SSG before and after each training period included the following: the percentage of successful individual passes, number of lost balls, frequency of individual duels, ball possession counts, interceptions, tackles, and headers. The SSGs were recorded using Sony Handycam DVD 850 video cameras positioned at an elevated vantage point along the halfway line, approximately 10 m behind the sideline. A hand notation system aligned with the technical definitions outlined in Table 1 was used to document these metrics. This approach has been validated as a reliable means of evaluating technical actions in soccer^18,19^. The selected technical actions were specifically relevant to the game, frequently occurring during SSGs and matches, and well-defined in the existing soccer literature^20,21^. An experienced researcher reviewed all SSG recordings, and each recording was analyzed twice to establish intrarater reliability using the kappa coefficient^15,22^. The kappa values for the assessed variables ranged from 0.86 to 0.94, indicating a high level of consistency in evaluations.

**Table 1:** Definitions of technical skills used in this study^19–21^.

Technical action	Definition
Pass	The act of a player in possession of the ball passing it to a teammate using various parts of the body (such as the foot, thigh, or chest) and employing different techniques, including ground passes, lobbed balls, chips, flicks, or volleys, over varying distances
Possession	The process of a player gaining or attempting to gain control of the ball and maintaining it within their team
Interception	Occurs when a player successfully contacts the ball, enabling them to gain possession and disrupt an opponent’s pass
Tackle	A defensive action aimed at dispossessing an opponent who currently controls the ball
Header	The action of a player using their head to make contact with the ball

#### Measurements of exercise intensity

Heart rate (HR) was continuously monitored at 5-s intervals during all training sessions using the Polar Team2 Pro System (Polar Electro OY), providing an objective measure of exercise intensity. HR data were expressed as a percentage of the maximum heart rate (%HRmax), which allowed for a thorough assessment of the physiological load. For each session, the average heart rate (HRmean) was computed for both HC and SSG training modalities. The %HRmax for each session was calculated using the following formula: %HRmax = (HRmean/HRmax) × 100. The internal training load was assessed immediately after each HC and SSG intervention using the Borg CR-10 scale (RPE)^23^, a widely recognized tool for measuring perceived exertion. This scale allows athletes to subjectively evaluate the intensity of their efforts, providing valuable insights into their psychophysiological responses to training. Previous research has validated its reliability and effectiveness in assessing exercise intensity across various sports contexts^24^. To ensure accurate and consistent responses, the participants were familiarized with the RPE scale. This familiarization phase included detailed explanations of the scale’s numerical values and verbal descriptors, along with practical applications to help athletes associate their perceived exertion with the corresponding RPE.

#### Physical enjoyment

To evaluate positive affect during physical activity, the Physical Activity Enjoyment Scale (PACES) was employed, consisting of eight items, as described by Selmi et al.^15^. Following each training session (5 min), participants responded to the question: “How did you feel during the exercise?” Enjoyment levels were measured on a 7-point scale, where 1 indicated a highly enjoyable experience and 7 indicated a lack of enjoyment. The total PACES score ranged from 8 to 56, with higher scores reflecting greater perceived enjoyment.

#### Statistical procedures

Statistical analyses were performed using the Statistical Package for the Social Sciences (SPSS, version 26.0; SPSS Inc., Chicago, IL, USA). Data are presented as mean (M) ± standard deviation (SD). Prior to applying parametric tests, the normality assumption was verified using the Kolmogorov–Smirnov test. The study employed a mixed design with repeated measures, including a between-subjects factor (group: SSG-G vs. HC-G) and a within-subjects factor (time: pre- vs. post-intervention). Accordingly, a two-way repeated-measures ANOVA (group × time) was conducted to examine the main and interaction effects on all physical performance (MAS, 10 m and 30 m sprint, five-jump test, Illinois agility test) and technical performance variables (duel, successful pass, lost ball, possession, interception, tackle, and heading). For the physiological variables HR (HRmean, %HRmax), RPE) and PACES score recorded during all training sessions were averaged per participant across the six-week intervention period, and group comparisons were performed using independent-samples Student’s t-tests, with effect sizes reported as Cohen’s d, with effect sizes classified as trivial (< 0.2), small (0.2–0.5), moderate (0.5–0.8), or large (> 0.8)^25^. Bonferroni-adjusted pairwise comparisons were applied as post hoc tests for significant ANOVA effects. Partial eta squared (η^2^_p_) was used to report effect sizes for ANOVA results. A significance level of *p* ≤ 0.05 was considered statistically significant.

## Results

### Physical performance

**Fig. 2:**
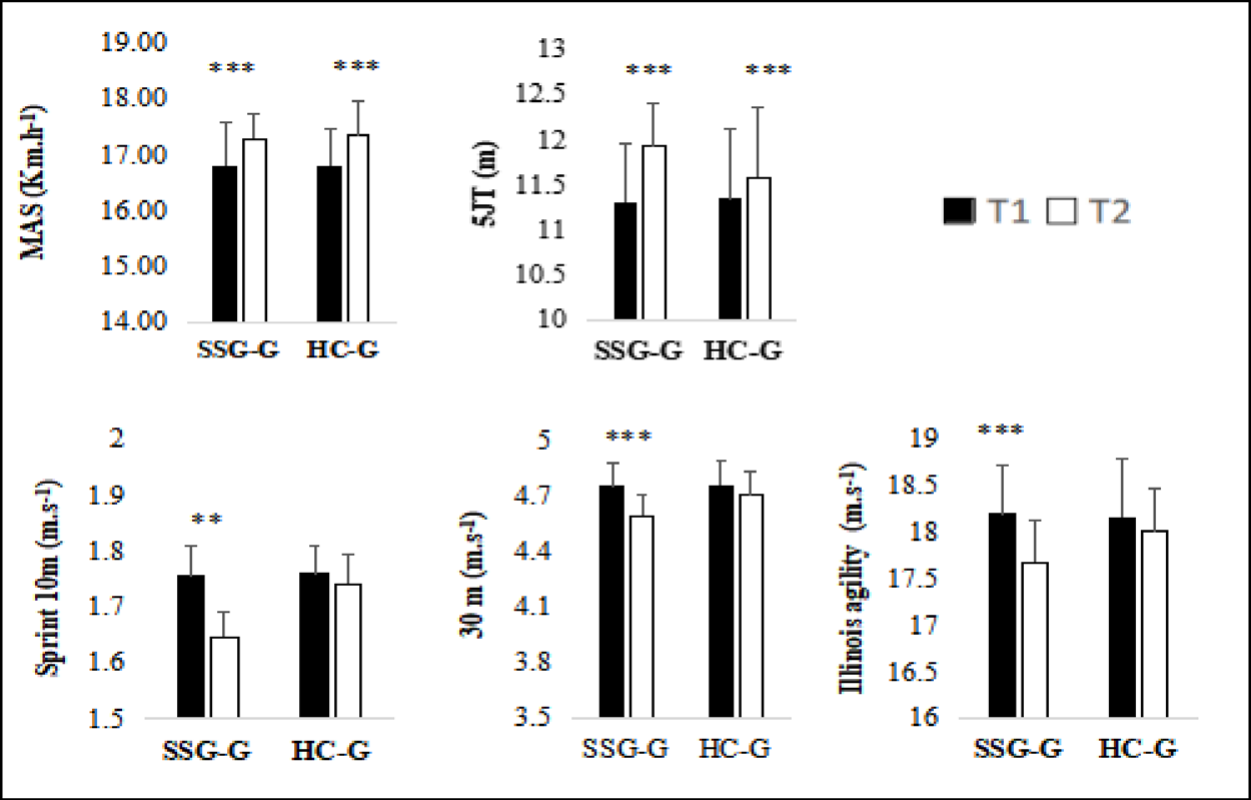
Physical performance measurements for both training programs (SSG and HC) before (T1) and after (T2) the 6-week intervention. MAS: Maximal Aerobic Speed; 10-m sprint time; 30 m: 30-m sprint time; 5JT: cumulative distance of five consecutive jumps; Illinois agility: time to complete the Illinois agility test; m: meters; km h^-1^: kilometers per hour; m s^-1^: meters per second. *Indicates a significant difference between pre- and post-intervention. **p < 0.01, ***p < 0.001.

Both training methods resulted in improvements in physical performance. Significant group × time interactions for sprint and agility measurements were found in the two-way repeated-measures ANOVA, along with significant main effects of time for MAS, 10 m and 30 m sprints, the 5-jump test, and the Illinois agility test. While both groups increased MAS and 5-jump performance, post-hoc comparisons revealed that SSG-G improved sprint times and agility more than HG (Table 2, Fig. 2).

**Table 2:** Two-way repeated-measures ANOVA results for physical performance variables.

Variables	Main effect of Group		Main effect of time		Interaction effect (Group × Time)	
	F	η2	F	η2	F	η2
MAS (Kmh^-1^)	0.24	0.01	32***	0.58	0.15	0.06
10 m (m s^-1^)	4.24*	0.15	7.05*	0.22	9.84**	0.29
30 m (m s^-1^)	1.50*	0.06	18.99***	0.44	4.53*	0.16
5 jump (m)	0.34	0.02	32.81***	0.57	5.95*	0.19
Illinois agility (m s^-1^)	0.54	0.02	14.01**	0.36	4.81*	0.17

Abbreviations: MAS: Maximum Aerobic Speed, 10 m: 10-m sprint speed, 30 m: 30-m sprint speed, 5 jump (m): cumulative distance of 5 jumps, Illinois agility: speed in the Illinois Agility Test, km h^-1^: kilometers per hour, m s^-1^: meters per second, m: meter.

*p < 0.05, **p < 0.01, ***p < 0.001.

### Technical performances

**Fig. 3:**
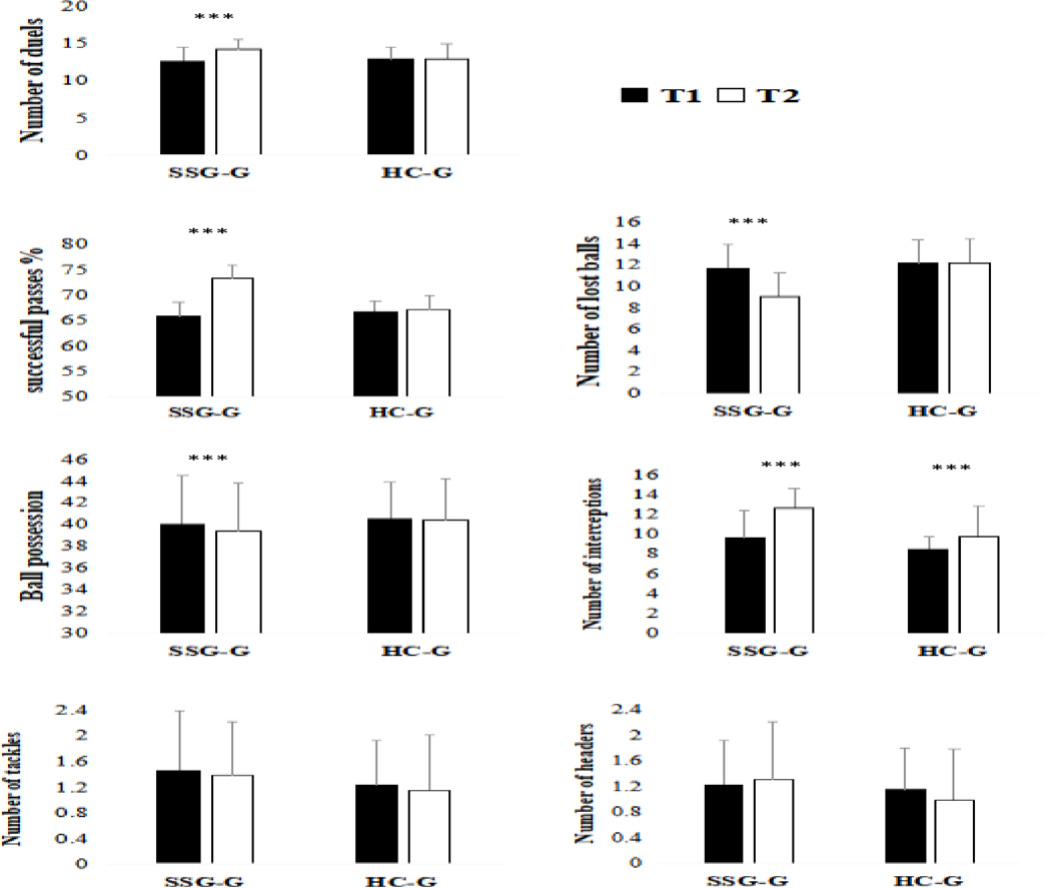
Technical aspect measurements for both training programs (SSG and HC) were taken before (T1) and after (T2) each intervention. SSG-G: small-sided games group; HC: Hoff circuit training group. Duel : number of duels won, %Successful pass : percentage of successful passes (%), Lost ball : number of balls lost, Possession : ball possession (%), Interception : number of interceptions, Tackle = number of tackles, Heading : number of successful headers. *Indicates a significant difference between pre- and post-intervention for both the SSG and HC training. ***p < 0.001.

Duels, successful passes, lost balls, and interceptions all showed notable increases with time, with significant group × time interactions, suggesting superior gains in SSG-G (Table 3). Only SSG-G showed significant improvements, according to post-hoc comparisons; HC-G showed no discernible changes. There were no discernible main or interaction effects for variables like possession, tackles, or headers (Fig. 3).

**Table 3:** Two-way repeated-measures ANOVA results for technical performance variables.

Variables	Main effect of the groupe		Main effect of the time		Interraction effect (Group × Time)	
	F	η2	F	η2	F	η2
Duel	0.74	0.03	16.85***	0.41	13.79**	0.36
%Succeful pass	7.57*	0.24	52.19***	0.68	44.32***	0.64
Lost ball	4.81*	0.16	17.95***	0.42	20.19***	0.45
Possession	0.24	0.01	0.44	0.02	0.21	0.009
Interception	5.04*	0.17	29.30***	0.55	4.52*	0.15
Tacle	0.46	0.02	0.31	0.01	0.000	0.000
Heading	0.43	0.02	0.11	0.004	0.96	0.03

Abreviations: Duel: number of duels won, %Successful pass: percentage of successful passes (%), Lost ball: number of balls lost, Possession: ball possession (%), Interception: number of interceptions, Tackle: number of tackles, Heading: number of successful headers, F: F-value, η^2^: effect size.

*p < 0.05, **p < 0.01, ***p < 0.001.

### The exercise intensity

No statistically significant differences were observed between SSG-G and HC-G in terms of RPE, HRmean, or %HRmax (*p* > 0.05) (Table 4).

**Table 4:** Comparaison of the psychophyiological variables between SSG-G and HC-G.

Variables	SSG-G	HC-G	95% cl	ES	Rating
RPE (UA)	5.9 ± 0.39	5.82 ± 0.42	-0.26 to 0.4	0.18	Trivial
HRmean (beats min^-1^)	175.9 ± 3.94	176 ± 5.11	-3.68 to 3.71	0.05	Trivial
%HRmax	84.91 ± 2.93	84.76 ± 4.03	-2.71 to 3.00	0.10	Trivial

Abbreviations: CI, confidence interval; ES, Cohen’s d effect size^25^; %HRmax, percentage of maximum heart rate; HRmean, mean heart rate; RPE, rating of perceived exertion; PACES, Physical Activity Enjoyment Scale; SSG-G, small-sided games group; HC-G, Hoff circuit training group. AU: Arbitrary Units; HRmean: Mean Heart Rate (beats per minute, bpm); %HRmax: Percentage of Maximal Heart Rate; ES: Effect Size; 95% CI: 95% Confidence Interval. Values represent averages per participant over the six-week intervention.

### Perceived enjoyment

**Fig. 4:**
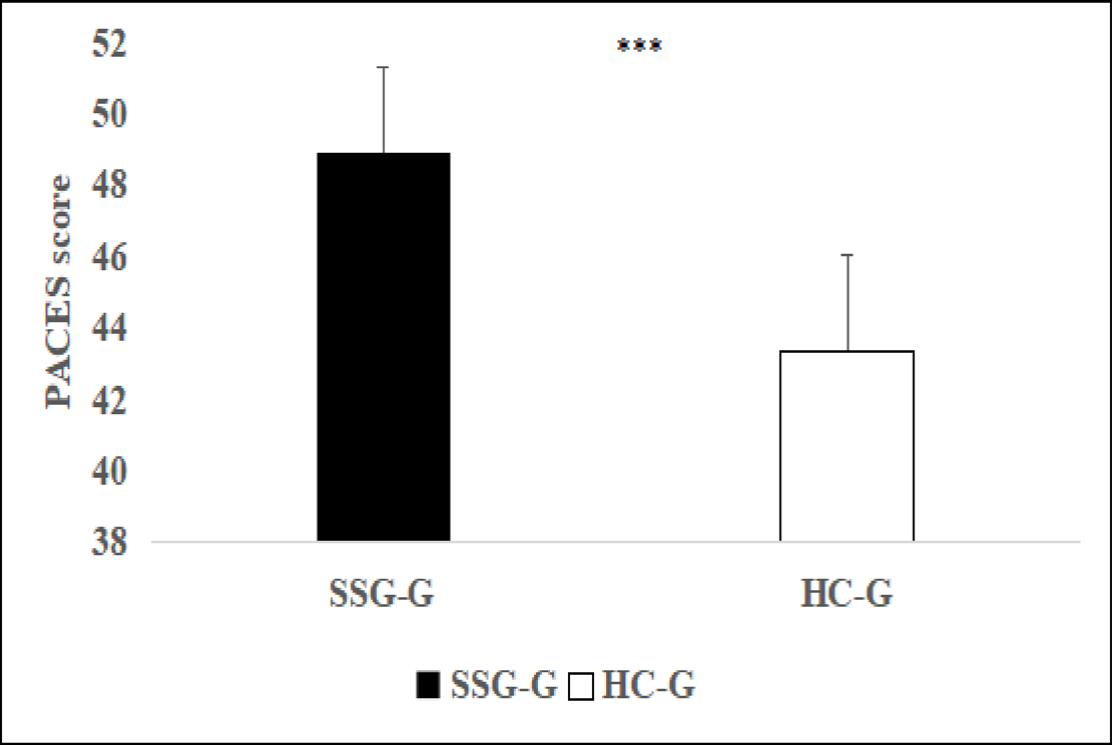
Comparison of Perceived Enjoyment between Training Programs. Values represent averages per participant over the six-week intervention. Abbreviations: PACES: Physical Activity Enjoyment Scale; SSG-G: small-sided games group; HC-G: Hoff circuit group. ***p < 0.001

Perceived enjoyment as measured by PACES, was significantly higher in the SSG-G (48.89 ± 2.45) than in the HC-G (43.37 ± 2.66; *p* < 0.001, ES = 2.55, large) (Fig. 4).

## Discussion

This study assessed the effects of SSG and HC training on the physical condition, technical aspects, physiological responses, and enjoyment of young soccer players. Both methods improved certain aspects of physical condition (e.g., MAS and 5-jump performance). However, only SSG training led to significant improvements in sprint times (10 m and 30 m), agility, explosive power, and technical skills (passing accuracy and interceptions). Additionally, the higher enjoyment associated with SSG suggests that it may lead to better long-term motivation and adherence than HC training.

Concerning the physical performance Our findings provide substantial evidence of improvements in multiple physical performance variables after both training programs. However, SSG-G demonstrated more pronounced improvements than HC-G, particularly in the 10 m and 30 m sprints, the Illinois agility test, and the 5-jump test.

The significant main effect of time observed for all physical performance measures, including the MAS, 10 m sprint, 30 m sprint, 5-jump test, and Illinois agility test, highlights the efficacy of both SSG and HC in enhancing physical performance. Notably, both training programs resulted in a significant increase in MAS (SSG: + 1.8 km h⁻^1^; HC: + 1.5 km h⁻^1^), indicating that both SSG and HC effectively improved aerobic capacity. These MAS gains are comparable to those documented in earlier research, which found that short high-intensity mesocycles resulted in MAS or VO₂/field-test improvements of a comparable size^2,8,26^. This supports the interpretation that over the course of six weeks, both procedures provided an aerobic stimulation strong enough to elicit central and peripheral adaptations. This is consistent with previous studies that have shown the benefits of high-intensity exercises like SSG^27–29^ and circuit training^6–8^ on aerobic fitness. The significant increase in MAS suggests that these programs can enhance the endurance capacity of soccer players, which is crucial for sustaining performance during match play^7,30^.

In terms of sprint performance, the 10 m and 30 m sprint times showed significant improvements in both groups, with the SSG-G exhibiting a more substantial reduction in sprint times. These results reflect the well-established benefits of SSG training in improving sprint performance, likely due to enhanced neuromuscular efficiency and sprinting mechanics^31,32^. The SSG-G demonstrated greater improvements in sprinting and explosive capabilities compared to the HC-G^30^. This finding is supported by the significant interaction effect between group and time observed in the 10 and 30 m sprint tests, which showed that the SSG-G group achieved greater improvements (10 m: F = 9.84, *p* < 0.01, η^2^ = 0.29; 30 m: F = 4.53, *p* < 0.05, η^2^ = 0.16). These results emphasize the potential of SSG in enhancing short-distance sprinting, which is a key component of soccer performance^31,33^. These findings are consistent with studies^30,31^, who found that game-based or match-profiled training produced better short-sprint and acceleration increases than more linear running or isolated conditioning. Moreover, the 5-jump test, which assesses explosive strength and lower-body power, also showed a significant increase in performance in both groups, with the SSG-G demonstrating a greater improvement^2^. This finding aligns with prior research suggesting that explosive strength can be enhanced through high-intensity interval training and sport-specific drills^34^. This result reinforces the notion that SSG, by incorporating repeated bouts of high-intensity activity, is particularly effective in enhancing power output and muscular endurance^2^. The Illinois agility test, which measures agility and quick directional changes, also exhibited significant improvements in both groups. However, as with the sprint and jump tests, the SSG-G showed superior results, particularly in terms of the time required to complete the test. This is consistent with the nature of SSG, which inherently involve frequent changes in direction, thus improving an athlete’s agility and ability to maneuver under pressure^31,35^. This result suggests that SSG plays a role in enhancing agility, which is an essential component of soccer performance, particularly in defensive and offensive maneuvers^36^. These results are in line with the experimental study^33^ and the meta-analysis by^34^, which showed that SSGs, as opposed to running-based HIIT or continuous conditioning, promote greater sport-specific adaptations, particularly in agility and explosive performance. Therefore, our results support the conclusion that SSGs provide a more efficient approach for developing neuromuscular and functional capacities that are directly relevant to the demands of team sport performance.

The results of this study highlight significant improvements in several key technical aspects of soccer performance following both SSG and HC training, with SSG showing greater effectiveness. higher gains in interceptions (+ 5.4% vs. + 1.8%) and passing accuracy (+ 7.3% in SSG-G vs. + 2.1% in HC-G) following SSG. These quantitative differences highlight how contextualised, high-pressure practice helps transfer technical skills to match-like situations^36^. For duels, a significant main effect of time was observed (F = 16.85, *p* < 0.001, η^2^ = 0.41), indicating overall improvement in players’ ability to win one-on-one challenges across the training period. Additionally, a significant interaction effect (group × time; F = 13.79, *p* < 0.01, η^2^ = 0.36) was found, suggesting that the magnitude of improvement differed between groups, with the SSG program leading to greater gains compared with the HC program. This improvement is likely due to the high intensity of both training programs, which simulate match conditions and emphasize physical confrontations for possession^4^. The most significant improvements were observed in the percentages of successful passes, lost balls, and interceptions. The SSG-G showed significant improvements in passing accuracy, likely due to the pressure-filled passing scenarios in SSGs, which replicate match situations and improve decision-making and technical skills^27^. Similarly, the SSG-G exhibited a significant reduction in the number of lost balls, reflecting better ball control and decision-making under pressure^27^. In addition, significant improvements in interceptions were observed in the SSG-G, suggesting that SSGs foster better defensive awareness and anticipation, which are key skills for intercepting passes^11^.

The psychophysiological responses to the SSG and HC training programs did not show any significant differences between the two groups in terms of RPE, mean HRmean, or %HRmax. These findings suggest that both training programs induced similar levels of exertion and physiological responses, as evidenced by the trivial effect sizes (ES) for these variables (RPE: 0.18, HRmean: 0.05, %HRmax: 0.10), with no statistically significant differences (*p* > 0.05). Both programs likely provided comparable intensity levels; therefore, they may be considered similar in terms of the physical demands placed on participants^9^. However, the perception of enjoyment was notably higher in the SSG-G than in the HC-G. This result indicates a strong preference for SSGs in terms of perceived enjoyment, confirming prior work by ^9^ showing that SSGs are perceived as more engaging, interactive, and motivating than structured conditioning. In line with ^15^, indicating that SSG might improve long-term engagement and training adherence. This suggests that the participants found the SSG to be more engaging and enjoyable than HC training, possibly because of the more interactive and game-like nature of SSGs, which may be perceived as more dynamic and fun^9^. This higher enjoyment may have important implications for participant adherence to training programs, as enjoyment is a key factor influencing long-term engagement and motivation^2,9,28^.

### Limitations

There may have been a number of limitations to this study because it was carried out during the competitive season. The results of physical, technical, and psychophysiological performance could have been affected by a number of factors, including the coach’s training regimen, match preparation, tactical plans, player rotation, and individual motivation. These factors should be taken into account when interpreting the results because they might have added variability beyond the training interventions themselves. By employing standardized team schedules or off-season interventions, future research could account for these variables.

## Conclusions

This study demonstrated that HC and SSG both successfully enhanced important facets of young soccer players’ physical performance. But when it came to improving sprint ability, agility, technical execution, and enjoyment, SSG training was more advantageous. SSG offers a more comprehensive and captivating method of player development by incorporating technical and physical challenges into realistic game environments. In contrast, on the other hand, HC is still useful for focusing on particular physical abilities in a regulated setting. All things considered, adding SSG to training regimens may maximize motivation, technical development, and athletic performance. Future studies should examine how both approaches might be integrated over time into periodized regimens and evaluate how player profiles affect their efficacy.

### Practical contribution

The results of this study suggest that incorporating SSG into training programs can provide significant benefits in terms of physical and technical performance for young soccer players. It is recommended that coaches prioritize SSGs due to their ability to enhance key skills such as speed, agility, explosive power, and passing accuracy, while creating a dynamic and engaging training environment. Furthermore, SSGs appear to offer a higher level of enjoyment, which could enhance long-term adherence to training. In addition, HC training can be used to target specific physical and technical attributes in a more controlled setting, such as aerobic endurance, repeated sprint capacity, and individual ball-handling skills. Therefore, designing training sessions that include SSGs for holistic, match-like development and HC for focused, isolated conditioning can optimize training effectiveness while maintaining player motivation and ensuring well-rounded athlete development.

## Data Availability

The data presented in this study are available upon request from the corresponding author. The data are not publicly available for further publication because of restrictions related to the ongoing work.

## Declarations

### Clinical trial registration

Clinical trial number is not applicable.
